# A newly isolated *Streptomyces nigra* strain for the biotechnological production of melanin

**DOI:** 10.1007/s00253-025-13673-1

**Published:** 2026-01-06

**Authors:** Donatella Cimini, Sergio D’ambrosio, Odile Francesca Restaino, Talayeh Kordjazi, Claudio Gervasi, Martina Aulitto, Islam Sayah, Paola Manini, Matilde Tancredi, Riccardo Peluso, Giuseppina Mandalari, Teresa Gervasi

**Affiliations:** 1 Department of Environmental, Biological and Pharmaceutical Sciences and Technologies, University of Campania Luigi Vanvitelli, Via Vivaldi 43, 81100 Caserta, Italy; 2 Department of Chemical Sciences, University of Naples Federico II, Monte Sant’Angelo Campus, Via Cintia 4, 80126 Naples, Italy; 3 Department of Chemical, Biological, Pharmaceutical and Environmental Sciences, University of Messina, Viale Ferdinando Stagno d’Alcontres 31, 98166 Messina, Italy; 4 Department of Biology, University of Naples Federico II, Monte Sant’Angelo Campus, Via Cintia 4, 80126 Naples, Italy; 5 Department of Biomedical Science and Morphofunctional and Functional Imaging, University of Messina, Viale Annunziata 38, 9816 Messina, Italy; 6 Research Unit UR17ES30 Genomics, Biotechnology and Antiviral Strategies, Higher Institute of Biotechnology of Monastir, University of Monastir, Tahar Hadded Avenue, PB74, 5000 Monastir, Tunisia

**Keywords:** Biotechnological production, Eumelanin, Genome mining, *Streptomyces nigra*, Whole genome sequence

## Abstract

**Abstract:**

Melanins are pigments widely distributed in microbial, plant, and animal kingdoms. Their UV–visible light shielding capacity, metal chelation ability, antioxidant, and antimicrobial properties make these pigments suitable for different industrial applications like in cosmetic and bioremediation fields. The actual manufacturing process relies on the extraction from animal tissues like the ink of *Sepia officinalis* and/or on synthetic chemical procedures. Streptomycetes might be the ideal candidates for the development of biotechnological processes of melanin production due to their ability to produce pigments as secondary metabolites, extracellularly released. Here, a new strain of *Streptomyces nigra,* capable of efficiently producing eumelanin, was isolated from soil samples in Messina, Sicily, Italy, and characterized first by 16S rRNA analysis and then by whole genome sequencing, with a complete gene clusters analysis. The strain ability of growing and producing melanin was tested on four media, including newly formulated ones, and by also optimizing temperature and pH conditions of growth, a melanin production of 2.45 ± 0.01 g/L was reached. The pigment, once produced under the optimal conditions, was purified and characterized by UV–visible, FT-IR, NMR, and EPR spectroscopy, revealing an eumelanin-like structure.

**Key points:**

• *A new Streptomyces nigra strain, MT6, was isolated and identified*

• *A new formulated medium boosted melanin production up to 2.45 g/L*

• *The extracellular pigment was characterized as eumelanin*

**Supplementary Information:**

The online version contains supplementary material available at 10.1007/s00253-025-13673-1.

## Introduction

Streptomycetes are Gram-positive, filamentous microorganisms characterized by a high guanine (G) and cytosine (C) content (G + C), spore-forming ability, and morphological features that resemble both bacteria and fungi (Javed et al. 2021). They represent valuable biotechnological microbial cell factories due to their ability to produce antibiotics (Mast and Stegmann 2019), to their capacity of synthesizing lignocellulosic enzymes useful in the degradation of organic matter in soil (Javed et al. 2021), and to produce different bioactive secondary metabolites and interesting pigments such as melanin (Selim et al. 2021). Melanin is a natural, polymeric pigment found across all biological kingdoms—bacteria, fungi, plants, and animals—and it is renowned for its broad-spectrum UV absorption, radical scavenging, antimicrobial and antioxidant properties, as well as anti-inflammatory properties (El-Naggar and El-Ewasy 2017; Kordjazi et al. 2024). These characteristics make melanin a highly attractive compound for several industrial sectors: in cosmetics and dermatology for sun protection and anti-aging formulations, in pharmaceutical field and biomedicine as a biocompatible component in drug delivery, as well as a radioprotective and antioxidant agent. Moreover, in environmental and technological fields, melanin is explored for heavy metal chelation, bioremediation, as a redox-active material and semiconductor in bioelectronic devices. In food and sustainable packaging sectors, melanin is incorporated into biodegradable films to enhance UV resistance, improve mechanical properties, and extend food shelf life (Tran-Ly et al. 2020; Tsouko et al. 2023). Despite its broad applicative potentials, the commercial use of melanin is limited since it is extracted from animal tissues such as the ink sac of *Sepia officinalis* or from plant materials. This kind of manufacturing process is labor-intensive, costly, and often environmentally and ethically problematic, as well as not sustainable. In addition, the pigment is often embedded in complex biological matrices, requiring harsh chemical treatments for purification, which can damage its structure and lower the yield (Pralea et al. 2019). Alternatively, melanin-like pigments might be chemically synthesized (Pralea et al. 2019). These issues have driven the scientific and industrial interest towards novel, alternative, and more sustainable manufacturing routes like microbial production, through the development of promising and scalable biotechnological processes. Numerous bacteria and fungi can naturally synthesize melanin, thus making microbial fermentation an alternative, more efficient, easy to scale up and environmentally friendly biotechnological process to produce the pigment (Kraseasintra et al. 2023). Among the several bacterial genera—including *Pseudomonas*, *Bacillus*, *Aeromonas*, and *Mycobacterium*—which have demonstrated melanogenic activity, *Streptomyces* spp. are among the most promising producers of melanin as they release it extracellularly during their secondary metabolism phase. *Streptomyces* strains synthesize eumelanin or pheomelanin (containing sulfur atoms) via a tyrosinase-mediated pathway. Tyrosinase is a monooxygenase with a di-nuclear copper catalytic center, which catalyses both the ortho-hydroxylation of monophenols and the oxidation of catechols transforming first L-tyrosine into L-DOPA and then L-DOPA into dopaquinone (Garcia-Molina et al. 2007). This compound might thereafter undergo further transformations and being converted into dopachrome and then into 5,6-dihydroxyindole-2-carboxylic acid (DHICA) or 5,6-dihydroxyindole (DHI) units that polymerize into eumelanin. In alternative pathway, dopaquinone, in the presence of cysteine or glutathione, forms benzothiazine that leads to pheomelanin polymerization (Ito & Wakamatsu 2003; Kordjazi et al. 2024). In *Streptomyces* species, the tyrosinase gene is part of the *melC* operon. Next to the tyrosinase gene (*melC2*), this operon also comprises an additional ORF called *melC1*, which is essential for the correct expression of the enzyme. The melanin biosynthetic genes *melC1* and *melC2* control the production and the secretion of the enzyme (Claus and Decker 2006). The expression of these genes depend also on environmental conditions, such as substrate and metal ion availability, that influence the type and the quantity of melanin produced by the different *Streptomyces *strains (Kordjazi et al. 2024). *Streptomyces*-derived melanins not only exhibit structural similarities to their animal counterparts but also play essential protective roles thanks to their numerous biological activities (El-Naggar and El-Ewasy 2017; Kordjazi et al. 2024). Given the versatility and established the biosynthetic capacities of *Streptomyces*, there is a considerable interest in identifying new strains with high melanin production potential and optimizing their cultivation conditions to enhance pigment yield and purity. In this study, the isolation and characterization of a novel *Streptomyces nigra *strain with strong melanogenic capacity is reported. The strain was characterized from a genomic point of view, and its pigment production potential was evaluated in initial physiological studies, also by formulating a new growth medium, and by testing different temperatures and pH conditions as well. The pigment, once produced under optimal conditions, was purified and characterized by UV–visible, FT-IR, NMR, and EPR spectroscopy.

## Materials and methods

### Soil sampling and isolation of Streptomycetes

Soil samples were collected at a depth of about 10–20 cm in soils in Messina, Sicily, Italy. The standard dilution plate method was used to isolate Streptomycetes strains. The isolation was conducted by using Petri plates containing starch nitrate agar medium, prepared with components purchased from Sigma-Aldrich (USA), with the following composition (g/L): starch, 20.0; KNO_3_, 2.0; K_2_HPO_4_, 1.0; MgSO_4_.7H_2_O, 0.5; NaCl, 0.5; CaCO_3_, 3.0; FeSO_4_.7H_2_O, 0.01; agar, 20.0; and distilled water up to 1 L; plates were then incubated for a period of 7 days at 30 °C (El-Naggar and El-Ewasy 2017). Representative colonies of Streptomycetes were picked up and repeatedly streaked on starch M-protein Agar (Himedia Laboratories, India), leading to the isolation of bacterial colonies.

### Screening for melanin producers

The newly isolated strains were screened by inoculating loopful of spores on peptone yeast extract iron agar plates (ISP medium 6), containing (g/L): peptone, 15.0; protease peptone, 5.0; ferric ammonium citrate, 0.5; K_2_HPO_4_, 1.0; sodium thiosulfate, 0.08; yeast extract, 1.0; and distilled water, 1 L; agar, 20.0 g; at pH 7.0–7.2 and on tyrosine agar (ISP medium 7) plates containing g/L: glycerol, 15.0; L-tyrosine, 0.5; L-asparagine, 0.5; K_2_HPO_4_, 0.5; MgSO_4_.7H_2_O, 0.5; FeSO_4_.7H_2_ O, 0.01; and distilled water, 1 L; agar, 20 g; at pH 7.2, according to previously reported methods (El-Naggar and El-Ewasy 2017). The agar plates were incubated at 30 °C for 6 days. Brown to the black zones of diffusible pigments around the colonies on the medium were registered as positive signals of a melanin producing strain.

### 16S rRNA based identification and phylogenetic analysis

The genomic DNA was extracted using the GeneJET Genomic DNA Purification Kit (Thermo Scientific, Milan, Italy) following the manufacturer’s instructions. The universal primer pair 27F (5′-AGAGTTTGATCMTGGCTCAG-3′) and 1492R (5′-TACGGYTACCTTGTTACGACTT-3′) were used, and Polymerase Chain Reaction (PCR) was performed using GoTaq® Colourless Master Mix (Promega, Madison, WI, USA) in a final volume of 50 µL. The PCR-amplified products were assessed on a 1.5% (w/v) agarose gel, and their concentration and purity were verified using an Implen N50 NanoPhotometer (Westlake Village, CA, USA). DNA sequencing of the purified fragments was performed by Macrogen Europe (Milan, Italy) using the same forward and reverse primers for bidirectional sequencing. The partial sequence of the 16S ribosomal RNA (rRNA) gene of strain MT6 was aligned with the corresponding 16S rRNA sequences of the type strains of representative members of the genus *Streptomyces* using the ClustalW algorithm (https://www.genome.jp/tools-bin/clustalw). The sequences were analyzed with the Basic Local Alignment Search Tool (BLAST, http://blast.ncbi.nlm.nih.gov/) to search for similarities against the National Center for Biotechnology Information (NCBI) database (https://blast.ncbi.nlm.nih.gov/Blast.cgi) and to calculate the statistical significance of the matches. The phylogenetic tree was constructed via the neighbor-joining method using MEGA 11.0 software (Tamura et al. 2021).

### Whole-genome sequencing and assembly

Genomic DNA was extracted from the isolated *Streptomyces* sp. by using the MasterPure™ Complete DNA Purification kit that allowed to obtain the quality required for further sequencing. The genome of the newly isolated *Streptomyces* was sequenced by using a 4th generation sequencing instrument the MiniIon, Oxford Nanopore. To construct the species tree, closely related genomes were selected from the RefSeq public repository via NCBI. This was done using the tools “Insert Genome Into SpeciesTree-v2.2.0” and “Build Phylogenetic Tree from MSA using FastTree2—v2.1.11” available in KBase. Genetic relatedness was further assessed by calculating the average nucleotide identity (ANI) usig FastANI v0.1.3, and the closest genome strains were identified. In addition, in silico DNA–DNA hybridization (DDH) values were calculated by using the Genome-to-Genome Distance Calculator (GGDC) v3.0. The genome sequence of *S. nigra* was assembled, annotated, and analyzed for orthologous groups, metabolic pathways, and secondary metabolites. The genome was annotated by using the NCBI Prokaryotic Genome Annotation Pipeline (PGAP) and Rapid Annotation of microbial genome using Subsystem Technology (RAST) version 2.0. The “Evolutionary genealogy of genes: Non-supervised Orthologous Groups” (EggNOG) database was used to predict and classify the clusters of orthologous groups (COG) associated with protein coding genes. The antisSMASH software version 7.1.0 was used to identify putative secondary metabolite biosynthesis gene clusters. Blastp (Basic Local Alignment Search tool) was used to analyze protein sequences for homology against the non-redundant protein database with default parameters (Altschul and Lipman 1990). The clusters potentially responsible for melanin biosynthesis were further analyzed with SignalP (Teufel et al. 2021) and DeepTMHMM (Hallgren et al. 2022) for the identification of signal peptides and transmembrane helices to better define protein localization (e.g., cytosolic, transmembrane, or extracellular). Furthermore, the protein family database (InterPro/Pfam) (Blum et al. 2025) was used to identify the family domains indicating the possible role of these proteins in the clusters. Finally, the Alphafold server (Abramson et al. 2024) was used for the generation of the predicted protein structures of MelC1 and MelC2 found in both clusters. These structures were then compared using the PDB Pairwise Structure Alignment tool (Bittrich et al. 2024) to identify the grade of structural homology.

### Microorganism propagation and media

The new strain was initially propagated from the isolation plates by inoculation in 50 mL tube containing 10 mL of GYA medium with the following composition (g/L): glucose, 20.0; yeast extract, 20.0; (NH_4_)_2_SO_4_, 2.0; NaH_2_PO_4_·H_2_O, 5.8; Na_2_HPO_4_, 8.2, at pH 7.0, 28 °C, and 250 rpm in a rotary air shaker (ISF-1-W, Kühner, Switzerland) for 48 h. Thereafter, this culture was used as inoculum in 1-L shake flask containing 200 mL of GYA medium; again, after 48 h of growth, the culture was centrifuged at 4 °C and 5000 rpm (Avanti J-20XPI, Beckman Coulter, USA) and then re-suspended in fresh GYA medium with 20% (v/v) glycerol to prepare cell stock solutions, then stored at −80 °C, according to previously reported procedures (Restaino et al.^2014^, ^2016^). All the medium components used in microorganism propagation and then in physiological studies were purchased from Himedia Laboratories (India), except for glucose, glycerol, (NH_4_)_2_SO_4_, NaH_2_PO_4_·H_2_O, Na_2_HPO_4_, and NaCl that were from Sigma-Aldrich (USA). For all the type of experiments, the media were sterilized by autoclaving at 120 °C for 20 min (ALFA-junior, PBI International, Italy). In case of glucose and Na_2_HPO_4_ presence as medium components, these two nutrients were added later to the media as solutions after being sterilized by filtration with 0.22 µm membranes (Millipore, France).

### Physiological studies for melanin production

In order to optimize the growth medium composition of the new isolated *Streptomyces nigra* MT6 strain for the melanin production, first physiological 250 mL-shake flask experiments were run in triplicate at 32 °C, pH 7.0, 250 rpm for 96 h on four different media: GEM III N, already previously reported (Restaino et al. 2014, 2016, 2020), containing (g/L) [GEM III N: glucose, 12.0; yeast extract, 6.0; malt extract, 30.0; NaH_2_PO_4_·H_2_O, 5.8; Na_2_HPO_4_, 8.2] and three new formulated media [SEM (g/L): rice starch, 12.0; yeast extract, 6.0; malt extract, 30.0; NaH_2_PO_4_·H_2_O, 5.8; Na_2_HPO_4_, 8.2; GSM (g/L): glucose, 12.0; soya peptone, 6.0; malt extract, 30.0; NaH_2_PO_4_·H_2_O, 5.8; Na_2_HPO_4_ (8.2 g/L); SSM (g/L): rice starch, 12.0; soya peptone, 6.0; malt extract, 30.0; NaH_2_PO_4_·H_2_O, 5.8; Na_2_HPO_4_, 8.2]. After medium optimization, growth conditions were tested by running in triplicate 250 mL-shake flask experiments at 250 rpm for 96 h at different temperatures in the range of 28–32 °C, and at pH 6.0 to 7.0. In all the experiments, samples of culture were withdrawn at different time points, every 24 h (0, 24, 48, 72, and 96 h), and used to determine the microbial growth and the produced melanin in the different conditions (Restaino et al. 2022, 2024). The determination of the biomass was performed by measuring the cell dry weight (in terms of g_cdw_/L) by filtering of 2.0 mL of broth samples on 0.22 µm polypropylene filters (Merck Millipore, France); the collected biomass was washed with physiological solution and then desiccated at 25 °C up to reach a constant dry weight (Restaino et al. 2022, 2024). Small volumes of the broth samples (2 mL) were also centrifuged at 4 °C and 5000 rpm for 20 min (Avanti J-20XPI, Beckman Coulter, USA) to obtain supernatant samples then used to determine the melanin concentration and the extracellular tyrosinase activity. In particular, the tyrosinase activity was assayed by adding 50 µL of each supernatant sample to a freshly prepared solution (950 µL) of 2.0 mM L-DOPA in 13.0 mM KH_2_ PO_4_ buffer at pH 6.5, and by following the reaction at 280 nm for 10 min at 25 °C (Spectrophotometer V-770, Jasco, Tokio, Japan), as previously described (Restaino et al. 2022, 2024, 2025). One unit of tyrosinase was defined as the amount of enzyme that gave an increase of 0.001 units of absorbance per minute (Masterman and Redding 2010). For the structural characterization of the pigment by the new strain, 1-L shake flasks were run in quadruplicate with 200 mL of SSM medium at pH 7.0 and at 30 °C, in a rotary air shaker (ISF-1-W, Kühner, Switzerland) at 250 rpm for 96 h, after inoculation with 400 µL of the bacterial cell stock solution. After the growths, the broths were centrifuged at 4 °C and 5000 rpm for 20 min (Avanti J-20XPI, Beckman Coulter, USA), and the collected supernatants were pooled together to be used to purify melanin.

### Purification of melanin

The melanin, extracellularly produced in the optimal conditions (SEM medium, 30 °C, pH 7.0), was purified from the clarified broth supernatant, after centrifugation at 4 °C, 5000 rpm for 20 min (Avanti J-20XPI, Beckman Coulter, USA), with an acid precipitation process by addition of 5.0 M HCl (Sigma-Aldrich, USA), up to a pH value of 1.5. The precipitated melanin was then further washed with the same acid solution under stirring conditions for 24 h and dried at room temperature to obtain a powder.

### Melanin characterization

The determination of the extracellular melanin concentration was performed on supernatant samples (2 mL) of clarified cultural broth, obtained after centrifugation at 4 °C and 5000 rpm for 20 min (Avanti J-20XPI, Beckman Coulter, USA), by UV measurements at 220 nm (Spectrophotometer V-530, Jasco, Japan), after building a calibration curve with the synthetic melanin used as a standard (Melanin Synthetic, cod. M863, Sigma-Aldrich, USA; no animal-derived melanin, like melanin from *Sepia officinalis*, cod. M2649, was available to be purchased and delivered by the same company to our lab at the moment when the experiments for this work started), in the range between 0.0005 and 0.01 g/L, as previously described (Restaino et al. 2022, 2024). Both the standard and the samples were diluted in a 0.1 M NaOH solution (Sigma-Aldrich, USA), also used as blank. To correctly determine the concentration of melanin produced by the microorganism, the initial absorbance value of the growth medium (0 h) was subtracted to the absorbance values of the different supernatants, as collected during the growth. After the production and the purification of the pigment by the new strain, the pureness of the pigment was evaluated dissolving a defined amount of the essiccated sample in a defined volume of 0.1 M NaOH. Besides, a complete UV–visible spectrum, in the range from 190 to 400 nm, was also acquired for the pigment, once purified. The standards and all the samples were always dissolved or diluted in 0.1 M NaOH solution, also used as blank. The purified melanin was also characterized by using FT-IR (Fourier-transform infrared spectroscopy), acquiring the spectra in triplicate in the range between 4000 and 700 cm^−1^, with 600 scans and a spectral resolution of 2.0 cm^−1^ (FT-IR-4700, Jasco, Japan), after preparation of a KBr (Sigma-Aldrich, USA) disk (200 mg) containing the pigment (1.2 mg). These FT-IR spectra were compared with the ones of the melanin standard (Sigma-Aldrich, USA) obtained in the same way. The purified pigment (33.2 mg) was also characterized by using NMR spectroscopy by using DMSO-*d6* as a solvent. ^1^H NMR spectra were recorded with a BrukerDRX-400 MHz instrument (Bruker Corporation, Billerica, MA, USA), whereas ^1^H,^1^H COSY and ^1^H,^13^C HSQC experiments were run at 400.1 MHz using standard pulse programs. Furthermore continuous-wave (CW) EPR spectra were recorded on a Bruker Elexsys E-500 X-band spectrometer operating at 9.87 GHz equipped with a high-sensitivity resonator. The powdered samples were introduced into sealed glass capillaries, which were then inserted into a 4 mm quartz sample tube for measurement. All spectra were acquired at room temperature under the following experimental conditions: sweep width = 140 G, data resolution = 1024 points, modulation amplitude = 1.0 G, conversion time = 20.5 ms, and time constant = 10.24 ms. Before recording, the modulation amplitude was optimized to prevent over-modulation, and the microwave power was adjusted to avoid distortion or premature saturation of the absorption signal. For power saturation experiments, the microwave power was systematically varied from 0.0008 to 98 mW. The evolution of the signal intensity with the square root of the applied power was then analyzed to assess the relaxation behavior of the paramagnetic centers. The peak-to-peak linewidth (ΔB) was determined directly from the first-derivative spectrum, while the g-factor and spin concentration were evaluated using an Mn^2^⁺-doped MgO internal standard, positioned within the same quartz tube. All measurements were repeated on independently prepared samples to ensure reproducibility, yielding estimated uncertainties of approximately 30% for radical concentration and 3 × 10⁻^4^ for the g-factor. The purified pigment was also tested for its antioxidant activity, evaluated by DPPH (1,1-diphenyl-2-picrylhydrazyl) assay, according to previously reported methods (Kordjazi et al. 2024). Briefly, different melanin solutions were prepared by dissolving the purified melanin in distilled water in a concentration range of 3–500 µg/mL. For each assay, the reaction mixture contains 100 µL of a 67.5 µmol/L solution of DPPH in methanol, with 10 µL of the different sample solutions and 190 µL of methanol in a 96-well microplate, in triplicate. The plate was incubated for 1 h at room temperature in dark conditions, and then the absorbance was then measured at 517 nm by using a microplate reader (Varian Cary 50 UV–visible spectrophotometer, USA). For the control sample, 10 µL of distilled water was used in replacement of the melanin sample, while for the blank sample, only methanol (290 µL) and 10 µL of distilled water were mixed. The percentage inhibition of radical scavenging activity was calculated using the following equation:

1 $$I\left(\%\right)\;=\;\left[\left(Ac-As\right)/Ac\right]\;\times\;100$$

where Ac was the absorbance of the control and As was the absorbance of the sample. The IC50 value, which represented the concentration at which 50.0% of DPPH radicals were neutralized, was determined from the concentration–response curves obtained by plotting the percentage of inhibition I (%) as a function of the concentrations of melanin solutions.

### Data analysis

All the data reported in the paper were average values of at least three independent experiments, calculated with their standard deviations by using a Microsoft Office Excel 2007 program (Microsoft, Redmond, Washington, DC, USA), while comparison between groups of data was performed with Student’s *t*-test and considered significantly different if *p* values were less than 0.05.

## Results

### *Streptomyces* isolation and molecular identification of melanin producing strains

**Fig. 1:**
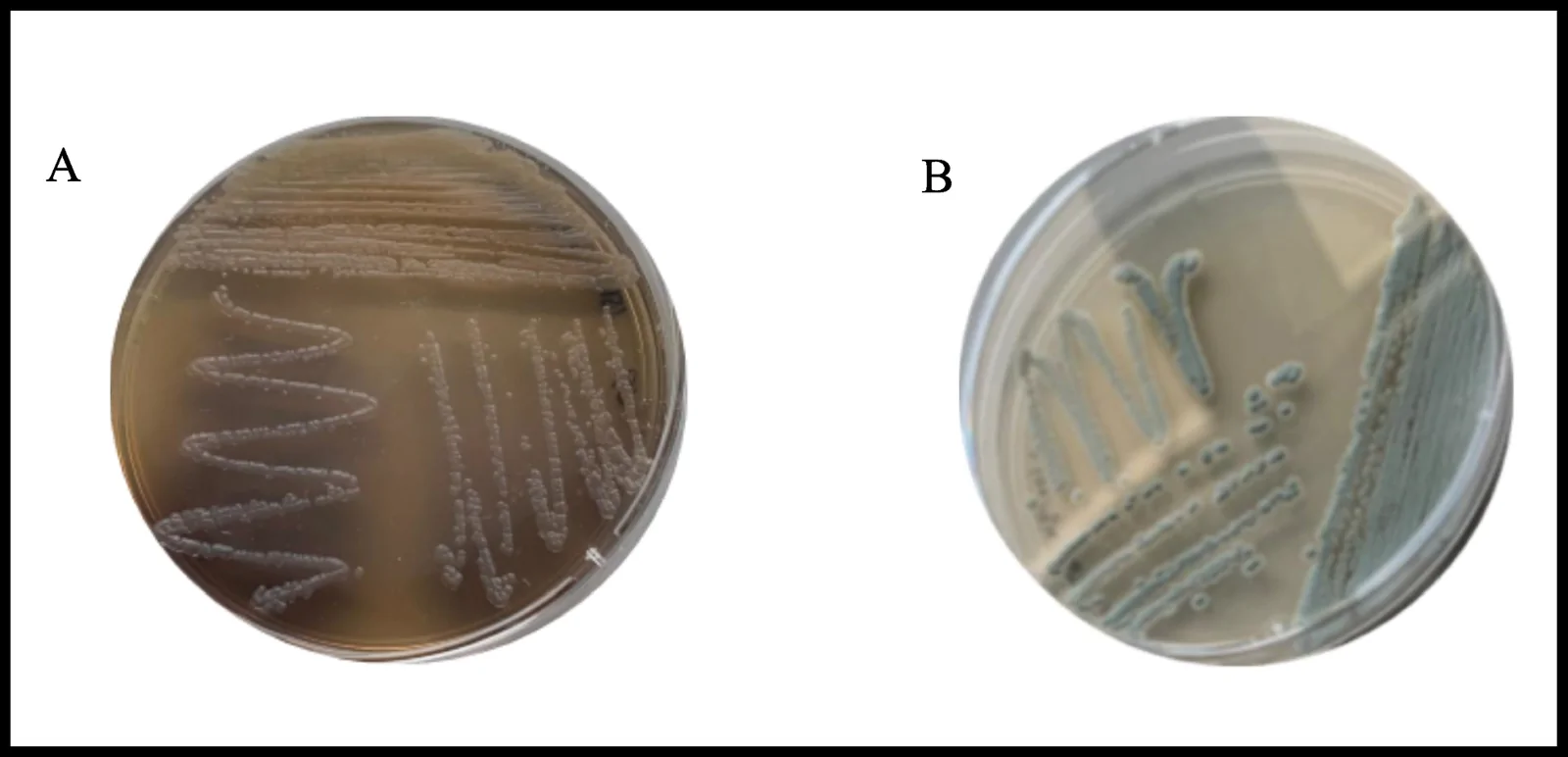
The new isolated strain grown on ISP6 (**A**) and ISP7 (**B**) media

**Fig. 2:**
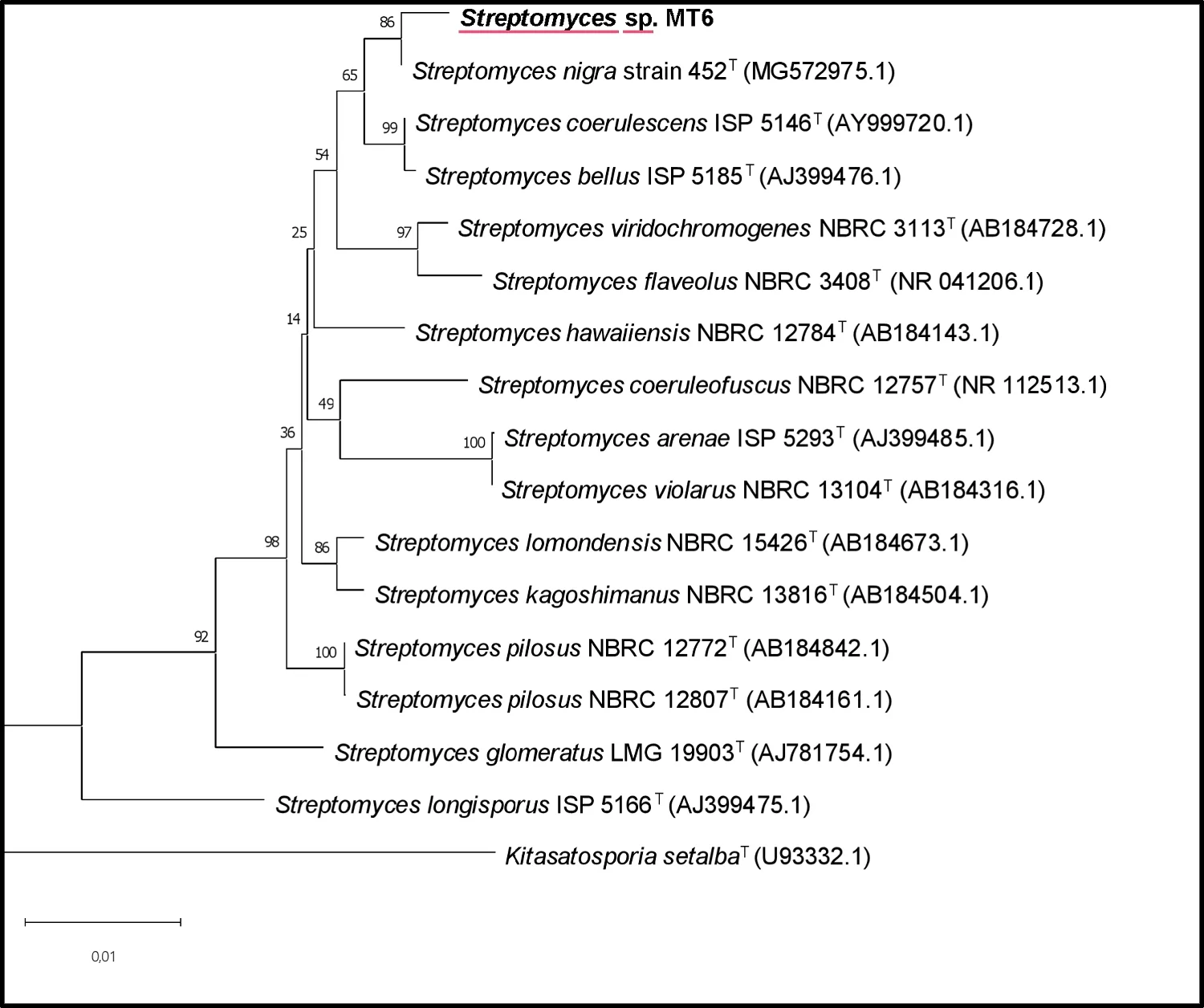
Neighbor-joining phylogenetic tree based on 1367 unambiguous nucleotides of the 16S rRNA gene sequence showing the position of the new isolated *Streptomyces nigra* strain among related species of the same genus. Their GenBank accession numbers are given in parentheses*. Kitasatosporia setalbaT* (U93332.1) was used as an outgroup. Numbers at the nodes indicate the percentages of bootstrap samplings. Bar, 0.01 substitutions per nucleotide position

A total of 50 different Actinomycetes strains were isolated from soil in Messina. The screening for the extracellular synthesis of melanin by all the isolates was performed, after the strain purification, on peptone yeast extract iron agar and tyrosine agar. The synthesis of melanin was assessed by the formation of brown zones around the colonies, and one strain was considered promising. The morphological characteristics of the newly isolated strain, as well as its production of melanin on ISP6 and ISP7 media, are shown in Fig. 1. The strain, designated MT6, showed a greyish aerial mycelium with faint brown substrate mycelium when grown on ISP6 and a dusty green aerial mycelium with brown substrate when grown on ISP7 (Fig. 1). Following the phenotypic selection, this strain was selected as potential microorganism for the synthesis of melanin, and its genetical and physiological characteristics were studied. The strain was initially called MT6. A BLAST search of the GenBank database using 1367 bp 16S rRNA gene sequence of strain MT6 showed a similarity between 98.0 and 100.0% to that of several species of the genus *Streptomyces.* In particular, the highest similarity was determined with *Streptomyces nigra* DICA-192 (99.56%), *Streptomyces nigra* TCA-065 (99.56%), *Streptomyces coeruleorubidus* 2–3–16 (99.56%), and *Streptomyces coeruleorubidus* T37 (99.56%), as well as > 99.49% similarity to other type species of the genu*s Streptomyces*. A phylogenetic tree (Fig. 2) based on 16S rRNA gene sequences of members of the genus *Streptomyces* was constructed using the neighbor-joining algorithm with bootstrap analysis according to Saitou and Nei (1987) with MEGA version 11 (Tamura et al. 2021). This tree showed the close phylogenetic association of strain MT6 with other *Streptomyces* species and highlighted the highest similarities within the *Streptomyces nigra* species, although species identification was then investigated by whole genome sequencing.

### Whole genome sequencing of *S. nigra* MT6

**Fig. 3:**
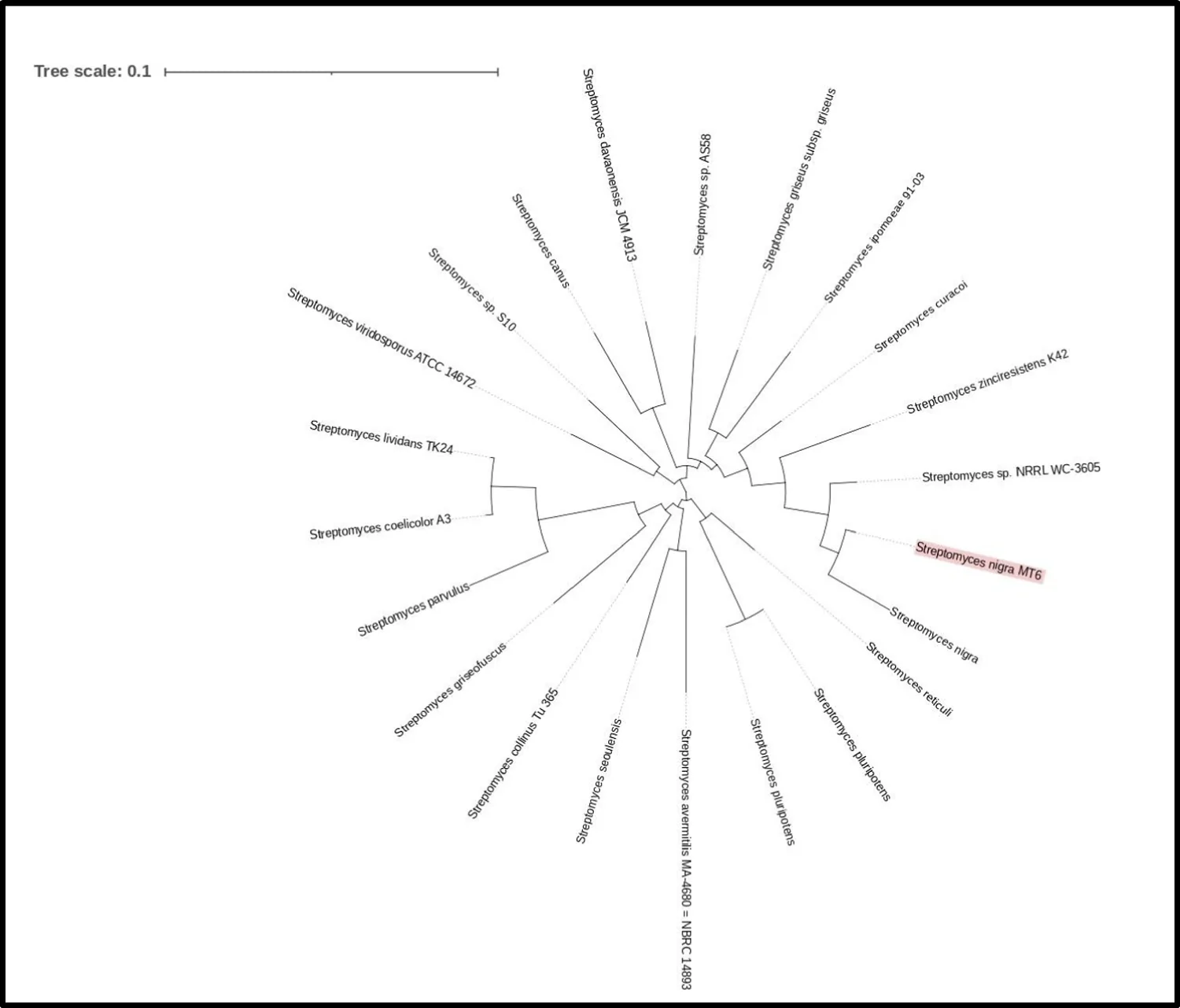
Phylogenetic tree based on the genome comparison of *S. nigra* MT6 and related species. The closest strains available in NCBI were used as reference genomes

**Fig. 4:**
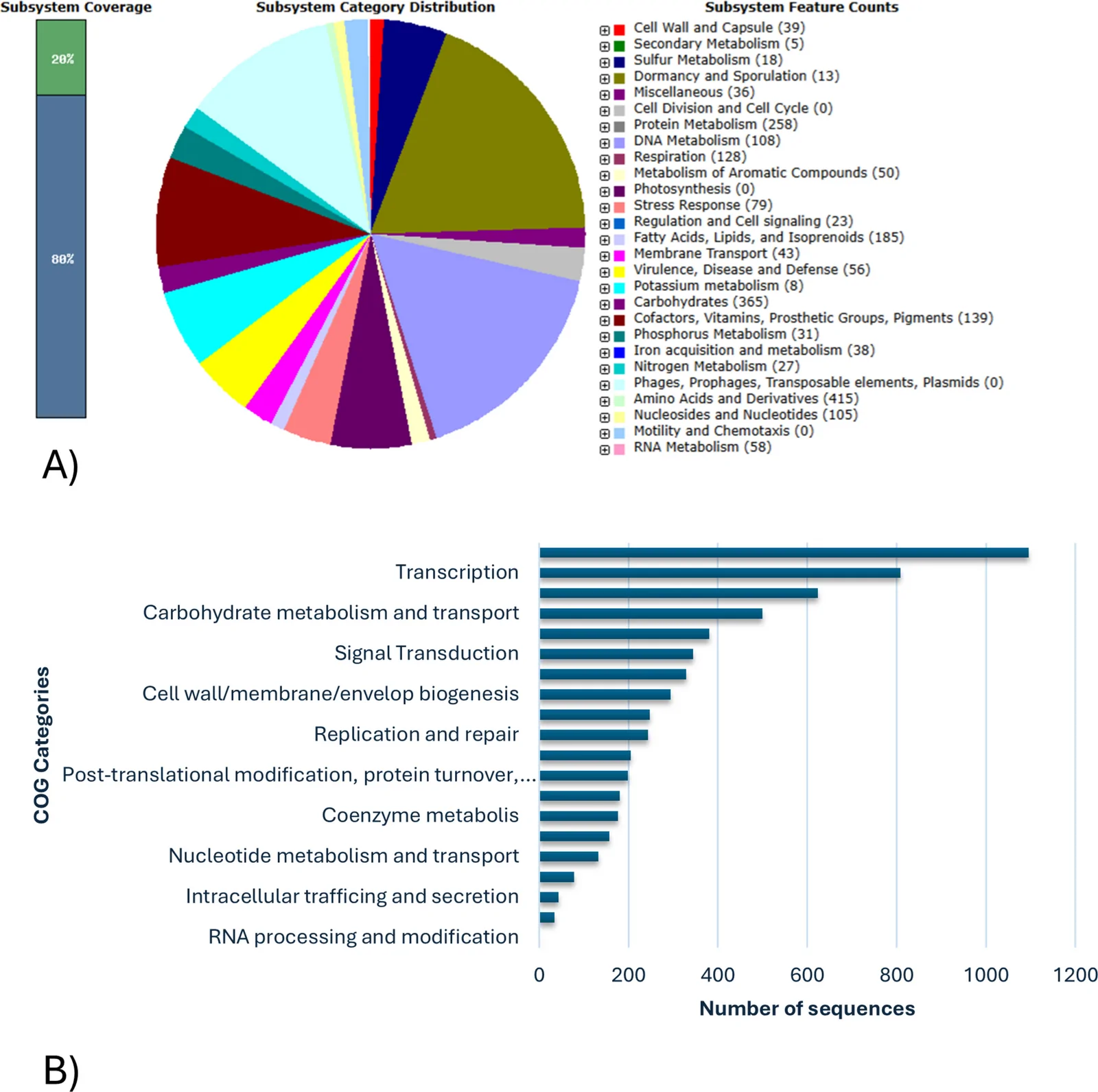
Subsystems distribution of the genome of *S. nigra* MT6 according to genome annotation with RAST (**A**); Clusters of Orthologous Groups (COG) distribution of the protein coding genes obtained from EggNog database (**B**)

Whole genome sequencing of the newly identified strain was performed for species identification and to identify potential gene clusters associated with melanin synthesis. Results are indicated in Fig. S1. A sequence of 7.741.885 bp was obtained, with an 87X coverage. The phylogenetic analysis confirmed *Streptomyces nigra* (GCF_003074055.1) as the closest species to the isolate (Fig. 3). The ANI estimated value between the new strain and the reference *S. nigra* 452 was 97.2%, exceeding the 95.0% threshold and thus confirming its assignment to the *S. nigra* species. DDH analysis further supported this species-level classification. Moreover, with the probability that DDH > 79.0% (indicating the same subspecies) being only 39.36%, the DDH results also confirm that the isolate is a new strain of *S. nigra*, designated *S. nigra* MT6. The whole genome sequence of *S. nigra* MT6 was assembled, annotated, and analyzed for orthologous groups, metabolic pathways, and secondary metabolites. The PGAP annotation showed 7031 CDs, of which 6255 coding genes with proteins and 781 pseudo genes, 88 RNA genes including 67 tRNAs, 18 rRNAs, and 3 ncRNAs. The whole genome sequence annotation details were reported in NCBI as BioProject PRJNA1162291, BioSample SAMN43807049, and gene bank accession number CP170373.1. Data collected from the RAST annotation server indicated that the draft genome contains 7501 coding sequences and 327 subsystems of which the most represented features are “Amino acids and derivatives” (415) and “Carbohydrates” (365) as indicated in Fig. 4A. The largest functional COG found by using the EggNog database belonged to the category of unknown proteins (1095) followed by those involved in transcription (808) and in amino acid metabolism and transport (624). Finally, 19 regions for putative secondary metabolite biosynthesis gene clusters were identified by using the antiSMASH tool, out of which two potentially responsible for melanin production (region 5 from 26744040 to 2684892 and region 15 from 6887168 to 6897584) as shown in Fig. 4B and Fig. S1. The search for similar known gene clusters within the antiSMASH database indicated a 4.0% similarity to istamycin for region 5 and a 57.0% similarity to the melanin biosynthesis cluster for region 15 (Fig. S2). The ORFs present in the two regions were characterized by using different bioinformatic tools (SignallP, DeepTMHMM, InterPro/Pfam), and results regarding protein length, homology, presence of signal peptide, and localization are reported in Fig. S2. Moreover, since a high difference in terms of similarity between the MelC1 and MelC2 proteins in the two clusters was observed, the Alphafold predicted structures were compared using PDB Pairwise Structure Alignment tool. As shown in Fig. S3A and B. the MelC1 proteins show a moderate structural similarity and probably the same fold (TM ~ 0.5), resulting in a conserved catalytic core. While for MelC2 proteins, high structural similarity (RMSD > 2.5 Å) is observed as well as the same fold (TM > 0.7), resulting in a strong homology and nearly full-length alignment.

### Physiological shake flask studies

**Fig. 5:**
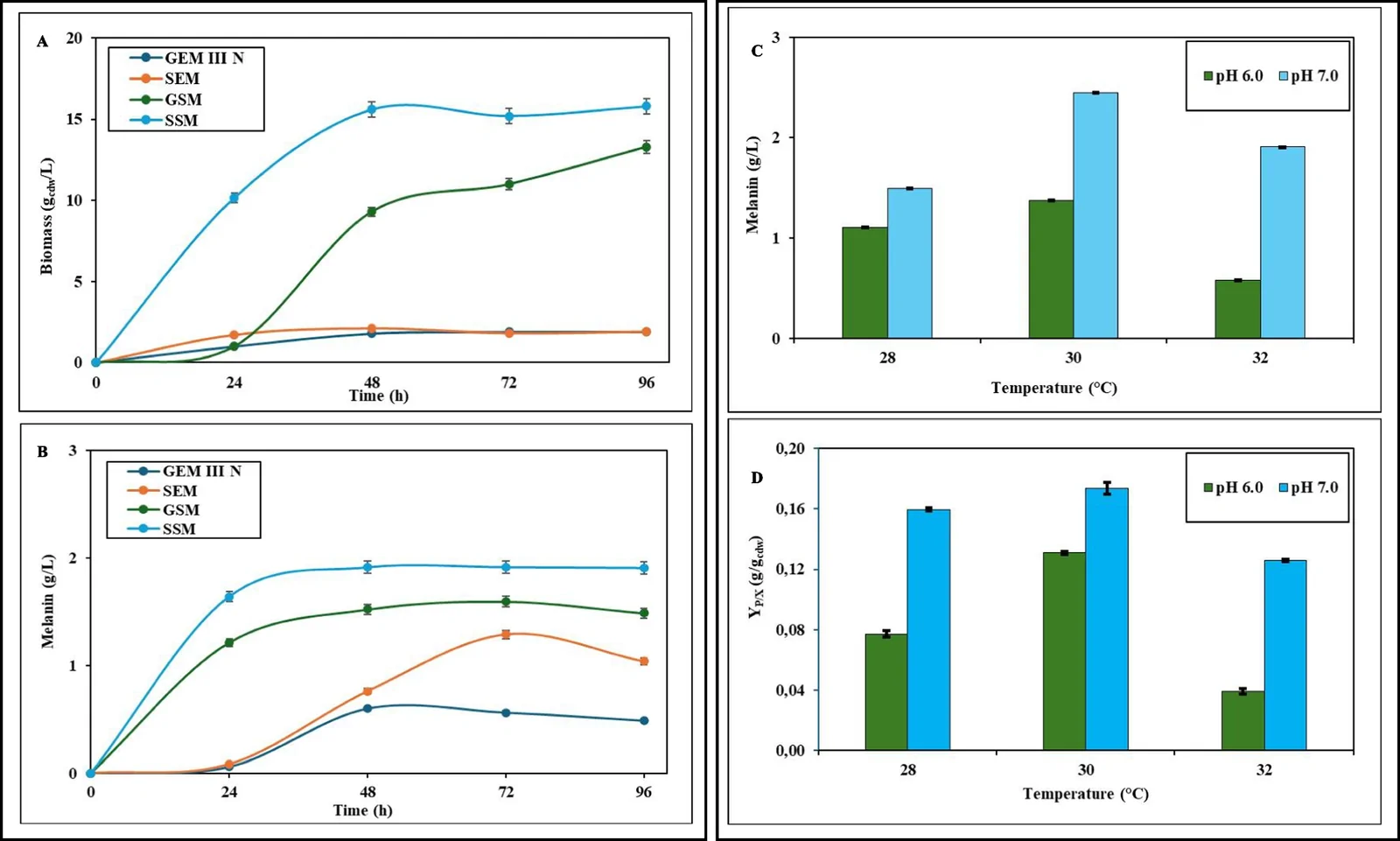
Growth curves (**A**) and melanin production (**B**) of the new isolated strain *S. nigra* MT6 on GEM III N, SEM, GSM and SSM media at 32 °C and pH 7.0. Melanin production (**C**) and melanin yield on biomass (**D**) on SSM media at different temperatures (28, 30, 32 °C) and pH (6.0 and 7.0) values. Statistical significance differences (*p* < 0.05) were found between the melanin production values and the yields on biomass at the two diverse pH conditions

Shake flask studies were performed to optimize the physiological conditions of growth and melanin production of the new isolated strain *Streptomyces nigra* MT6. Initially, to optimize the medium composition, runs were performed for 96 h, at pH 7.0 and 32 °C by using a medium already known (GEM III N) (Restaino et al. 2014) and three new *ad hoc* formulated different media (SEM, GSM, SSM). Data showed an influence of medium components on both biomass and melanin production: in fact, taking GEM III N medium as the reference one, 8.2 and 3.4 higher values, respectively, were noted on SSM medium by substituting glucose with rice starch and yeast extract with soya peptone (Fig. 5A, B). On SSM medium, a maximum biomass value of 15.6 ± 0.5 g_cdw_/L and a melanin production of 1.91 ± 0.01 g/L were reached, while on GEM III N medium, a maximum biomass value of 1.9 ± 0.1 g_cdw_/L (Fig. 5A**)** and a melanin production of 0.56 ± 0.02 g/L were obtained (Fig. 5B). The values obtained on SSM medium were the highest ones, compared also with the data obtained on SEM and GSM media. Thus, the SSM medium was considered the optimal one for the melanin production by *Streptomyces nigra* MT6 and then further studies were performed testing different pH values (6.0 or 7.0) and temperatures (28, 30 or 32 °C) to set up the best physiological conditions. Data showed that a further 1.3 increased melanin production, up to 2.45 ± 0.01 g/L, with a yield on biomass of 0.17 ± 0.01 g/g_cdw_, was reached by changing the temperature at 30 °C, while keeping the pH at 7.0 (Fig. 5C, D). In these optimal conditions, the extracellular tyrosinase activity showed a maximum of 3.2 U/mL at 72 h (Fig. S4).

### Purification and characterization of melanin

**Fig. 6:**
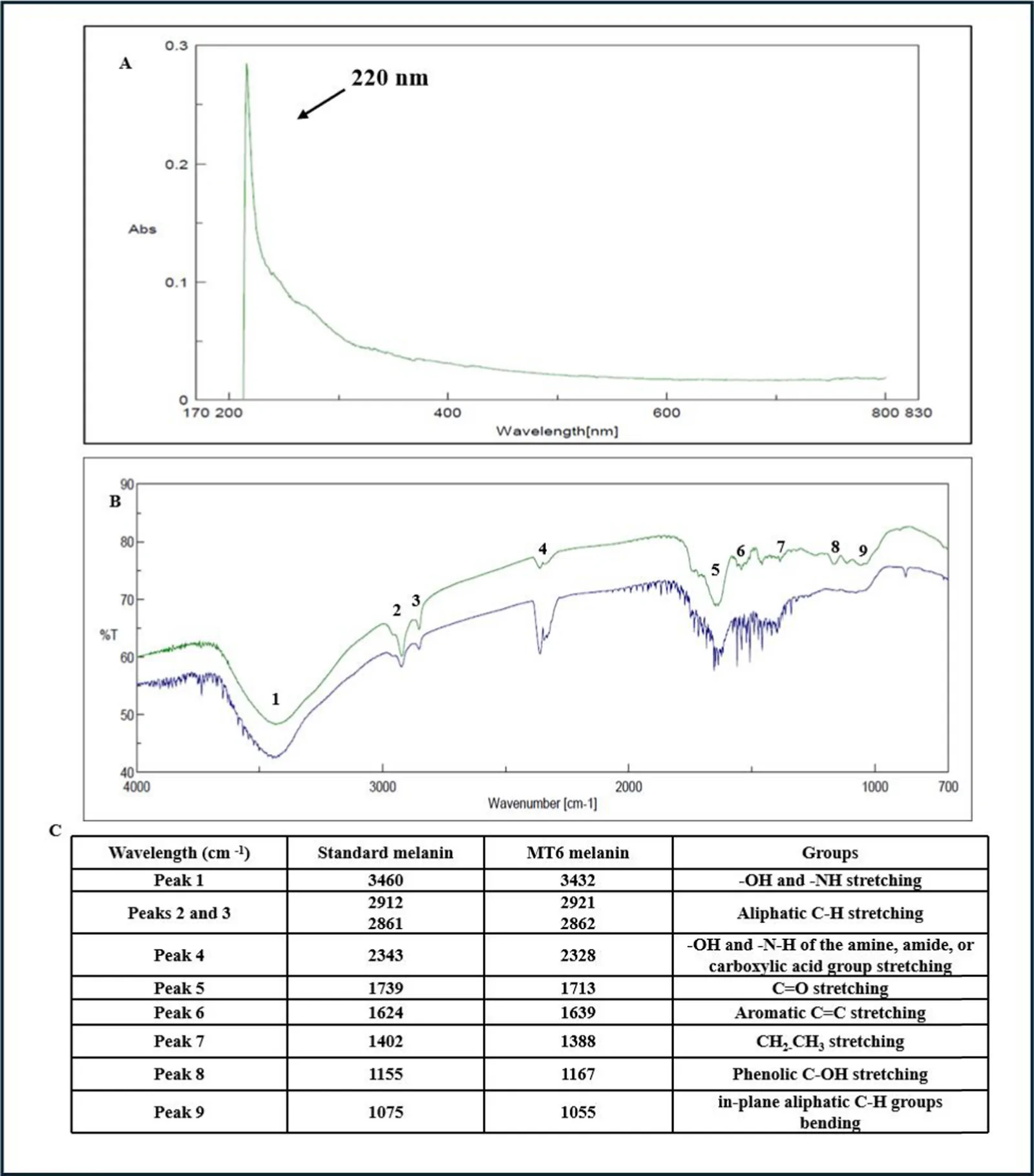
UV–visible spectrum of the purified melanin by *S. nigra* MT6 (**A**), with a maximum peak at 220 nm as indicated by the arrow in the comparison of the FT-IR spectra (**B**), with the peak table (**C**), of the purified melanin (green line) and of the synthetic melanin standard (blue line)

**Fig. 7:**
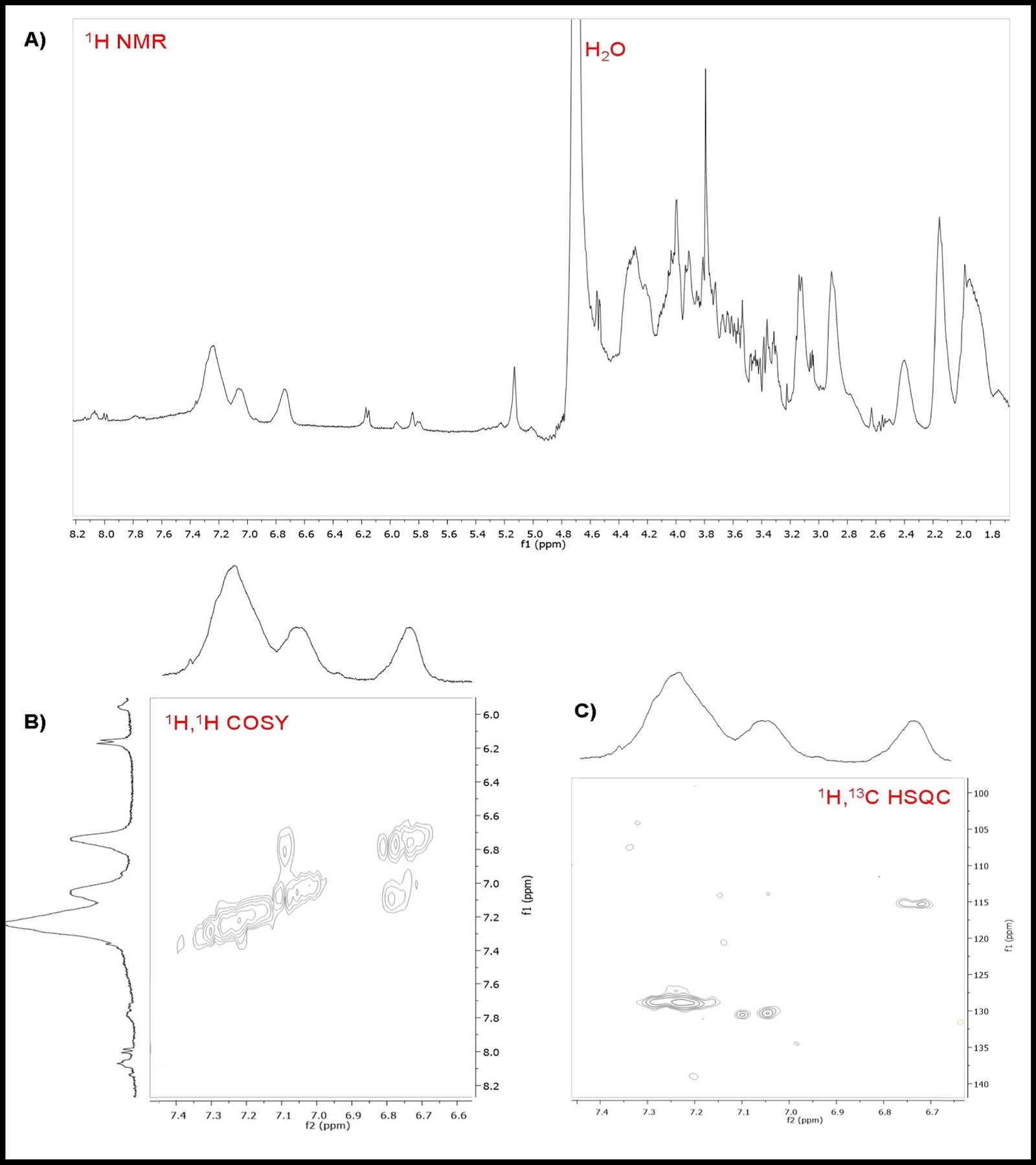
Mono-(^1^H) (**A**) and bidimensional ^1^H,^1^H COSY (**B**) and ^1^H,^13^C HSQC (**C**) NMR spectra of the purified melanin pigment produced by *S. nigra* MT6

**Fig. 8:**
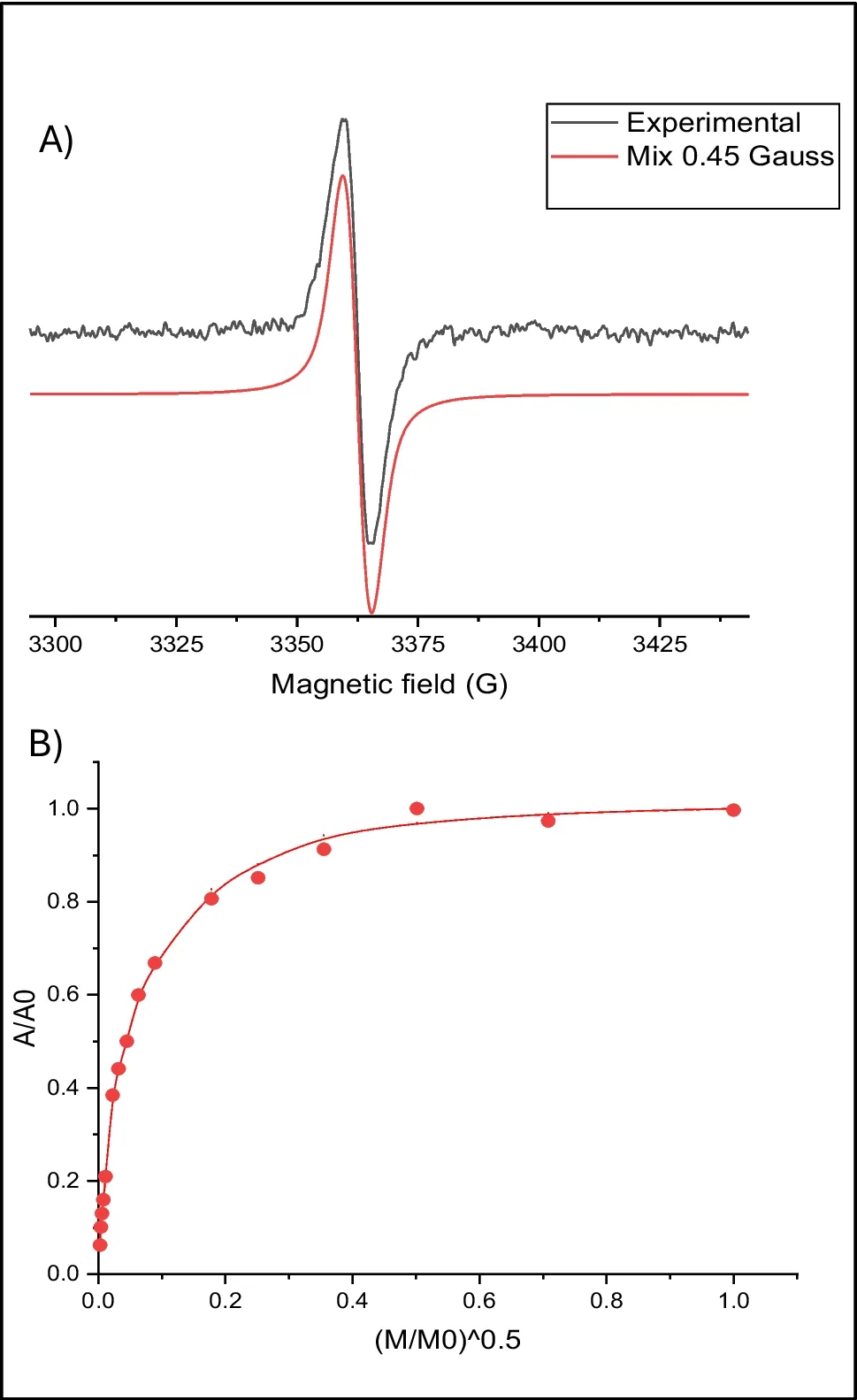
EPR spectrum (black trace) along with the best fitting (red trace) (**A**) and power saturation profiles (amplitude vs power intensity) (**B**) of the melanin pigment purified by *S. nigra* MT6

The new formulated and optimized SSM medium was considered suitable to produce melanin by the new strain, and it was used in 1 L-shake flask growths to produce enough amount of melanin to be then purified and characterized. After 96 h of growth, the clarified broth supernatant was recovered, and the melanin was precipitated by adding an acidic solution, up to pH 1.5, as already published (Restaino et al. 2022, 2024, 2025). The precipitated melanin was further purified by acidic washes and finally desiccated. The purification process allowed to recover about 70.0% of the produced melanin, with a purity grade of about 80.0%. The purified melanin was then characterized by UV–visible, FT-IR (Fig. 6A–C), NMR (Fig. 7A–C), and EPR spectroscopy (Fig. 8 and Table 1). In the UV–visible spectrum, the purified melanin showed the characteristic absorption profile of eumelanin with the typical monotonic decay and a maximum peak at 220 nm with a gentle slope towards the visible region, as previously reported data (Restaino et al. 2022, 2024, 2025; Kordjazi et al. 2024) (Fig. 6A). FT-IR analysis of the purified sample showed nine signals, specific to the melanin pigment, most of them similar to peaks previously reported for eumelanin structures (Restaino et al. 2022, 2024, 2025; Kordjazi et al. 2024) (Fig. 6B, C). A strong, broad peak centered at 3432 cm^−1^ showed the stretching of -OH and -NH groups of the indolic units (Peak 1), while the small peaks (Peaks 2 and 3) at 2921 and 2862 cm^−1^ were attributed to the stretching vibration of aliphatic C-H groups. Signal at 2328 cm^−1^ (Peak 4) were due to the stretching vibrations of the amine, amide, or carboxylic acid groups of the indolic units. Signal at 1713 cm^−1^ (Peak 5) was attributed to the C = O stretching of quinone or carboxylic acid groups, while the peak at 1639 cm^−1^ (Peak 6) was usually due to the stretching of the aromatic C = C groups. The peak at 1388 cm^−1^ (Peak 7) was usually considered characteristic of the melanin pigments and due to the -CH_2_-CH_3_ bending. The small peak at 1167 cm^−1^ (Peak 8) was due to the stretching of phenolic groups while the signal at 1055 cm^−1^ (Peak 9) was due to the bending of in-plane aliphatic CH groups. The purified melanin was also characterized by mono- and bidimensional NMR spectroscopy (Fig. 7A–C). As shown in Fig. 7A, in the aromatic protons region, three main broad peaks centered at 6.71, 7.07, and 7.24 ppm are clearly visible and ascribable to the H-3, H-4, H-7, and H-2 protons of the 5,6-dihydroxyindole nucleus. This assignment was further supported by ^1^H,^1^H COSY (Fig. 7B) showing the scalar coupling between the H-3 signal at 6.71 ppm and the H-2 signal at 7.07 ppm and by the ^1^H,^13^C HSQC spectrum (Fig. 7C) assigning the signals at 115, 128, and 131 ppm to C-3, C-4/C-7, and C-2, respectively, in good agreement with literature data (d’Ischia et al 2022). Finally, to further address unambiguously the melanin nature of the pigment purified by *S. nigra* MT6, EPR spectroscopy was employed. As widely reported in the literature (Panzella et al. 2013), melanin is characterized by a persistent paramagnetic signal due to the presence of unpaired electrons delocalized over the polymer backbone. As shown in Fig. 8, the pigment purified by *S. nigra* MT6 also exhibited an intense EPR signal characterized by a g-factor value (2.0038), a band width (5.4 Gauss), and a spin density (1.4E + 20 spin/g) comparable with those obtained from other eumelanin-like pigments as the melanin pigments extracted from *Sepia officinalis* and from black sturgeon caviar (Panzella et al. 2022). The purified melanin also demonstrated to have an anti-oxidant activity with an IC50 value of 12.5 ± 0.1 µg/mL.

**Table 1:** EPR parameters of the melanin pigment purified by *S. nigra* MT6 in comparison with other pigments of animal and synthetic origins

Melanin	g-factor	Δ*B* (Gauss)	Spin density (spin/g)
Melanin by *S. nigra* MT6	2.0038	5.4	1.4E + 20
*Sepia* melanin	2.0039	4.8	5.9E + 18
Melanin from black sturgeon caviar	2.0037	5.2	7.1E + 15
DHI-melanin	2.0038	4.7	1.3E + 21
DHICA-melanin	2.0038	4.3	1.6E + 21
DHN-melanin	2.0038	4.7	1.1E + 23
DOPA-melanin	2.0038	4.7	8.6E + 20

## Discussion

Streptomycetes are promising microbial cell factories for the biotechnological production of melanin pigments. In this perspective, many efforts have been made in the last years to discover new, efficient melanin producing strains, especially the ones able to synthesize eumelanin whose structure resembles the mammalian pigment (Kordjazi et al. 2024). In this paper, a wide research was initially performed to isolate a new *Streptomyces* strain from soil samples of Messina, Sicily, Italy. Among the 50 screened isolates, only 4.0% demonstrated the ability to produce melanin on selective media. These findings are consistent with previous studies, which reported that only a small fraction of Actinomycetes isolated from various ecological niches possess the capability to synthesize melanin. In particular, for members of the *Streptomyces* genus, the percentage of melanin-producing strains was reported to be less than 10.0% (El-Naggar and El-Ewasy 2017). For example, Shaaban et al. (2013) reported that among 30 microbial isolates obtained from Egyptian soil, only 3.0% demonstrated melanin production. Similarly, Dastager et al. (2006) found that among 180 *Streptomyces* isolates collected in the Gulbarga region, just 5.0% produced a diffusible dark brown pigment on selective media. El-Naggar and El-Ewasy (2017) also observed that among 130 Actinomycetes strains isolated from different localities in Egypt and Saudi Arabia, only 9.0% could produce melanin. The new isolate MT6 exhibited the typical characteristics of the genus *Streptomyces* based on its taxonomic properties, and the phylogenetic analysis of 16S rDNA sequence further supported its affiliation to the genus. As shown in the neighbor-joining phylogenetic tree, the strain MT6 clearly clustered into the clade of the genus *Strepomyces* tree, with all reference from *Streptomyces* showing more than 99.0% similarity to that of strain MT6. As a matter of fact, the most closely related species were *Streptomyces nigra* DICA-192 (99.56%), *Streptomyces nigra* TCA-065 (99.56%), *Streptomyces coeruleorubidus* 2–3−16 (99.56%), and *Streptomyces coeruleorubidus* T37 (99.56%). The 16S rRNA gene sequence is widely used as a molecular marker for bacterial taxonomy and phylogeny due to its universal presence and conserved nature among prokaryotes (Weisburg et al. 1991). Sequence similarity thresholds have been proposed to support taxonomic classification, with 97.0% similarity commonly used to delineate bacterial species (Stackebrandt and Goebel 1994) and 94.5% similarity as a tentative cut-off for genus-level differentiation (Yarza et al. 2014). However, despite its widespread use, the 16S rRNA gene exhibits notable limitations when applied to the genus *Streptomyces*. This is primarily due to its highly conserved nature, which fails to resolve closely related species within the genus (Law et al 2018). For all these reasons, further whole genome analyses were performed to confirm the identity of the new strain. The availability and constant advances of next generation sequencing technologies give the opportunity to better characterize newly identified strains. In this respect, whole genome analysis was carried out, and ANI and DDH values demonstrated the identification of a new isolate belonging to the *Streptomyces nigra* species. *Streptomyces nigra* is a recently identified species, first described by Chen et al. (2018) as sp. nov., isolated from mangrove soil and named for its dark, melanin-rich colonies. Genome size and the percentage of G + C of MT6 were in range with average values found for other Streptomycetes (Lee et al. 2020). In order to check for the presence of genes involved in melanin biosynthesis and to consider the strain potential to produce secondary metabolites, the antiSMASH tool was used. Indeed, it was recently reported that strain-level genome sequencing can reveal significant differences among biosynthetic gene clusters of strains that are associated to the same species, thereby uncovering novel compounds or derivatives (Belknap et al. 2020). AntiSMASH provided the detection of 19 gene clusters for secondary metabolites identifying two regions potentially responsible for melanin-type compounds based on the software database. Several literature studies demonstrated that different *Streptomyces* species possess multiple melanin operons; Oyserman and coworkers identified two melanin BGCs attributed to *Streptomyces* in the same strain associated with the tomato rhizosphere (Oyserman et al. 2022). Omura and collaborators (2001) found four gene clusters related to the melanin biosynthesis in the genome of an industrial strain of *S. avermitilis*, two of which encode the tyrosinase and its cofactor. In *S. nigra* MT6, region 15 showed a higher similarity with biosynthetic gene clusters responsible for melanin biosynthesis, while region 5 showed 4.0% similarity with known clusters responsible for istamycin biosynthesis. The same results were obtained by analyzing the genome of the reference strain *S. nigra* 452, while results from the antiSMASH analysis of *S. cavouren*sis, also known for melanin production, indicated that region 5 was categorized as responsible for melanin-, non ribosomal peptide synthetase (NRPS)-, and NRP metallophore-type compounds with similarity to coelichelin. ClusterBlast analysis of *S. nigra* MT6 region 5 and region 15 identified several homologous gene clusters across published genome sequences with major similarities with other *S. nigra* strains, namely strain RK62 and strain 452, as expected. Both regions contain *melC1* and *melC2* genes coding for the chaperone and tyrosinase enzyme, respectively, that are necessary for melanin production. To better characterize putative enzymes from the two potential melanin biosynthetic clusters, different bioinformatic approaches were applied. Besides those encoding for MelC1 and MelC2, some interesting ORFs were found in both regions. Region 5 was found to contain ORF 2536 possessing a domain present in transcriptional activators that may possibly bind DNA, as indicated by structural modelling (Rigden 2011). ORF 2539 is a transmembrane protein with five transmembrane domains that also contains a vitamin K epoxide reductase domain, which can be related to the redox/copper homeostasis or cofactor regeneration; finally, ORF 2540 (glycoside hydrolase family 16 protein) is an extracellular enzyme that degrades polysaccharides, and potential glycoside hydrolases have been found in clusters responsible for the biosynthesis of other secondary metabolites produced by *Streptomyces* (Carlson et al. 2010; Gomez et al. 2012). Region 15 seems to present a more complete configuration designed for both biosynthesis and export of melanin or its precursors. In fact, besides the *melC1/melC2* operon (ORF 6422 and 6423), the cluster includes several redox enzymes like FAD-dependent monooxygenase (ORF 6424), Zn‑dependent ADH (ORF 6419), aldo/keto reductase (ORF 6425), LLM/F420 oxidoreductase (ORF 6427), and a VOC family protein (ORF 6420). An extracellular SGNH/GDSL hydrolase (ORF 6418) is also present, and it may be involved in secondary metabolite secretion through its role in cell surface modification. Interestingly, ORF 6421 represents an RNase HI protein. The presence of this gene within region 15 may indicate an integrated response to stress damage. RNase HI is implicated in the removal of RNA:DNA hybrids (R-loops) induced by replicative or transcriptional stress (Krishnan et al. 2016), and the *Streptomyces* literature shows that biosynthetic clusters are often located near DNA repair or stress-management genes (Caicedo-Montoya et al. 2021; Du et al. 2024). In environments in which melanin is produced as defense from ROS/UV, an adjacent RNase HI gene could facilitate genome protection from simultaneous internal damage. The two different *melC1* and *melC2* ORFs from regions 5 and 15 were further characterized also in comparison to *S. nigra* strains RK62 and 452. Interestingly, Blastp comparisons highlighted a high identity (99.0–100.0%) of MelC2 proteins within the three strains, whereas the identity of the tyrosinases among the two clusters (within each strain) is reduced to 52.0–55.0% on average. To deepen this aspect, the Alphafold predicted structures of the two proteins were compared indicating for MelC1 a conserved catalytic core, while for MelC2, high structural similarity was confirmed. These results demonstrate a highly probable direct evolutionary homology of these proteins despite the different percentage of sequence identity. *S. nigra* 452 was previously reported to secrete melanin (Chen et al. 2018). However, studies regarding the investigation and optimization of melanin production were not conducted on any of the mentioned strains yet. Wise physiological studies might be performed with new isolated *Streptomyces* strains, melanin producers, in order to enhance the pigment production. As a matter of fact, as other metabolites produced by Streptomycetes, melanin synthesis is greatly dependent on the growth conditions, like pH, temperature, oxygen availability, and on medium composition in terms of types of carbon and nitrogen sources, on the presence of precursors, like L-tyrosine, or of co-factors like copper or iron (Barbuto Ferraiuolo et al. 2021; Wang et al. 2019; Kordjazi et al. 2024; Restaino et al. 2024, 2025). Complex substrates are usually employed for the growth of Streptomycetes (Table S1). Soluble starch (10.0 g/L) was used together with casein (0.30 g/L) in case of *S*. BJZ10 to reach up to 3.0 g/L of melanin in 120 h shake flask growths (Kazi et al. 2022), while peptone (15.0 g/L) and proteose peptone (5.0 g/L) were used together in the same medium to obtain 0.35 g/L of melanin in 168 h shake flask growths of *S. glaucensens* NEAE-H (Table S1). In this paper, the new formulated medium contained, for the first time, rice starch, while other components were, instead, already previously employed, like the soya peptone used in the medium of *Streptomyces* sp. ZL-24 (Wang et al. 2019) or the yeast extract and the malt extract present in *S. roseochromogenes* ATCC 13400 and *S. nashvillensis* DSM 40314 media (Restaino et al. 2024), allowing to reach melanin production values of about 4.24 g/L, 3.94 g/L, and 0.74 g/L, respectively (Table S1). Besides, in our studies, also the optimal pH and the temperature conditions for both the growth and melanin production were investigated in ranges commonly explored for this genus (Kordjazi et al. 2024). Both parameters resulted critical for melanin biosynthesis, and a pH value of 7.0 and a temperature of 30 °C resulted the optimal parameters, similar to the conditions found for *Streptomyces nashvillensis* DSM 40314 (pH 7.0 and 28 °C) (Restaino et al. 2024) and *Streptomyces* sp. ZL-24 (pH 7.0 and 30 °C) (Wang et al. 2019). This optimization studies allowed to reach a maximum of melanin production of about 2.45 g/L in the range of concentrations previously reported for *S*. BJZ10 (Kazi et al. 2022), *Streptomyces nashvillensis* DSM 40314 (Restaino et al. 2024), and *Streptomyces* sp. ZL-24 (Wang et al. 2019). Once produced in the optimal conditions, the pigment was purified by acidic precipitation and washes following a procedure already reported that assured high recovery and pureness values as previously found for the melanin produced by *S. roseochromogenes* ATCC 13400 (Restaino et al. 2022) and *Streptomyces nashvillensis* DSM 40314 (Restaino et al. 2024). UV analysis showed a spectrum with the typical monotonic decay curve with a maximum of absorbance at 220 nm, like those already reported for pigments produced by *Streptomyces lusitanus* DMZ-3 (Madhusudhan et al. 2014), *Streptomyces glaucescens* NEAE-H (El-Naggar and El-Ewasy 2017), and *Streptomyces* sp. ZL-24 (Wang et al. 2019). FT-IR analyses showed signals typical of a eumelanin-like pigment and similar to what already registered for *Streptomyces glaucescens* NEAE-H (El-Naggar and El-Ewasy 2017), *Streptomyces* sp. ZL-24 (Wang et al. 2019), and *Streptomyces nashvillensis* DSM 40314 (Restaino et al. 2024). Other similarities emerged from the inspection of the 1D/2D NMR spectra revealing the presence of signals in the aromatic protons region quite similar those observed in the case of other melanins produced by *Streptomyces roseochromogenes* ATTC 13400 (Restaino et al. 2022) and S*treptomyces nashvillensis* DSM 40314 (Restaino et al. 2024), suggesting the eumelanin nature of the pigment. The purified pigment also showed a moderate DPPH antioxidant activity whose value fits in a range between the 28% data reported for *Streptomyces cavourensis* RD8 (Dholakiya et al. 2017) and the 56% for *Streptomyces* sp. ZL-24 (Wang et al. 2019).

## Conclusions

In this study, a novel strain of *Streptomyces nigra*, MT6, was successfully isolated from sicilian soil and characterized for its capacity to produce eumelanin. Phylogenetic and genomic analyses confirmed its identity as a new strain within the *Streptomyces nigra* species. Genome mining revealed two distinct gene clusters potentially involved in melanin biosynthesis, including the presence of the essential *melC1* and *melC2* genes. Physiological optimization studies identified SSM medium, and pH 7.0 and 30 °C, as the most favorable conditions for growth and pigment production, to achieve a melanin concentration of 2.45 g/L, with a yield of 0.17 g/g_cdw_. The pigment was purified and characterized by UV–visible, FT-IR, EPR and NMR analyses, confirming its eumelanin-like structure. This work highlights the potential of *Streptomyces nigra* MT6 as a microbial platform for sustainable melanin production, offering an environmentally friendly alternative to traditional extraction or synthetic routes. The results lay a solid foundation for further bioprocess development, scale-up, and functional testing of the pigment in industrial applications such as bioplastics, cosmeceuticals, and environmental remediation.

## Supplementary Information

Below is the link to the electronic supplementary material.

ESM 1 (912 KB DOCX)

## Data Availability

The strain whole genome sequence has been deposited in NCBI (The annotation details are BioProject: PRJNA1162291, BioSample: SAMN43807049 accession number CP170373.1). The strain *S. nigra* MT6 is available from the corresponding author upon reasonable request. The data presented in this study are available from the corresponding author upon reasonable request.
