# Supplementary material for: A newly isolated Streptomyces nigra strain for the biotechnological production of melanin
Source: Appl Microbiol Biotechnol. 2026 Jan 6;110:4. doi: 10.1007/s00253-025-13673-1 (PMC12779669; doi:10.1007/s00253-025-13673-1)
Supplement: Supplementary file 1 [file 253_2025_13673_MOESM1_ESM.docx]

**A newly isolated *Streptomyces nigra* strain for the biotechnological production of melanin**

Donatella Cimini, Sergio D’ambrosio, Odile Francesca Restaino*, Talayeh Kordjazi, Claudio Gervasi, Martina Aulitto, Islam Sayah, Paola Manini, Riccardo Peluso, Giuseppina Mandalari, Teresa Gervasi.

*Corresponding author: O. F. Restaino, Department of Chemical Sciences, University of Naples Federico II, Monte Sant’Angelo Campus, via Cintia 4, 80126, Naples, Italy. e-mail: odilefrancesca.restaino@unina.it. Phone: +39812539473; ORCID 0000-0001-5939-3759.

**Submitted to Applied Microbiology and Biotechnology**

Genome coverage:

**Fig. S1** Bacterial genome summary report generated through the wf-bacterial-genomes workflow provided by Oxford Nanopore Technologies

**Fig. S2** Bioinformatic analysis of region 5 and region 15 identified from antiSMASH as potentially responsible for melanin production showing the predicted genes and functional annotations in Blasp and localization in SignalP and DeepTMHMM. Sec/SPI: Sec translocon/Signal Peptidase I; Tat/SPI: Tat translocon/Signal Peptidase I.

A

B

Fig. S3. PDB results of structural similarity between MelC1 and MelC2 in the two clusters. RMSD: root mean square deviation TM-score: template modeling score (A). Alphafold structures of MelC1 and MelC2 from region 5 (brown) and region 15 (blue) aligned with PDB Pairwise Structure Alignment tool (B).

**Fig. S4** Extracellular tyrosinase activity of *S. nigra* MT6 on SSM medium during a 96-h shake flask growth at 30 °C and pH 7.0

**Table S1** Melanin production by different *Streptomyces* strains by using diverse media and growth conditions
